# Association of prior interstitial lung disease awareness with knowledge, attitudes, and practices in Saudi Arabia: a national cross-sectional study

**DOI:** 10.3389/fpubh.2026.1830926

**Published:** 2026-07-20

**Authors:** Malik A. Althobiani, Husam I. Alahmadi, Fatma Almaghlouth, Turky Zafir, Ali Alshamrani, Ziyad Alalawi, Mazen M. Homoud, Hanin Alghamdi, Heba M. Bintalib, Mouaid Aljehani, Abdulalrahman Alharbi, Jaber S. Alqahtani, Abdullah Sakkat, Hajed M. Al-Otaibi, Abdullah A. Alqarni, Anne-Marie Russell

**Affiliations:** 1 Department of Respiratory Therapy, Faculty of Medical Rehabilitation Sciences, King Abdulaziz University, Jeddah, Saudi Arabia; 2 UCL Respiratory, University College London, London, United Kingdom; 3 Ministry of the National Guard - Health Affairs, Jeddah, Saudi Arabia; 4 Department of Respiratory Care, Prince Sultan Military College of Health Sciences, Dammam, Saudi Arabia; 5 Department of Medicine, King Abdulaziz University, Jeddah, Saudi Arabia; 6 School of Health Sciences, University of Birmingham, Birmingham, United Kingdom

**Keywords:** practices, attitudes, diagnostic delay, interstitial lung disease, knowledge, public awareness

## Abstract

**Introduction:**

Interstitial lung diseases (ILDs) are a heterogeneous group of diffuse parenchymal lung disorders, including idiopathic interstitial pneumonias, connective-tissue-disease–associated ILD and sarcoidosis. Patients often experience prolonged diagnostic delays and non-specific symptom profiles that overlap with more prevalent conditions. While these delays are well documented, the contribution of low public awareness to these delays is not well described in the Middle East. We aimed to determine the prevalence of ILD awareness in Saudi Arabia and evaluate its independent association with knowledge, attitudes, and care-seeking practices.

**Methods:**

We conducted a cross-sectional study of adults (≥18 years) across Saudi Arabia between May and October 2025. The study was reported in accordance with the Strengthening the Reporting of Observational Studies in Epidemiology guideline (STROBE). Data were collected using a validated knowledge, attitudes, and practices questionnaire, developed with reference to World Health Organisation guidance and the Theory of Planned Behaviour. Factors associated with prior ILD awareness were assessed using multivariable logistic regression. KAP domain scores, Knowledge (0–8), Attitude (1–5), and Practice (1–5) were compared between awareness groups using Mann–Whitney U tests and analysed using multivariable linear regression with standard errors, adjusting for demographics and healthcare background. *p* Values were adjusted for multiple comparisons using the Benjamini Hochberg false discovery rate procedure. Ethical approval was obtained from King Abdulaziz University.

**Findings:**

Of 830 individuals who initiated the survey, 628 (75.7%) provided complete responses and were included in the analysis. Of 628 participants, 116 (18.5%) reported prior ILD awareness. Healthcare providers had 6.45-fold higher odds of awareness (95% CI 3.12–13.32; *p* < 0.001), and other demographic factors showed no independent association. After adjustment, prior awareness was associated with higher knowledge (*β* = 3.27, 95% CI 2.77–3.77; *p* < 0.001) and higher practice scores (*p* < 0.001). Attitude differences were small and were not statistically significant after full adjustment (*p* = 0.051). Lack of awareness was reported as a care-seeking barrier by 81% (413/512) of unaware and even by 78% (90/116) of aware participants. Unaware participants prioritised symptom information (65% vs. 53%), whereas aware participants prioritised specialist centre access (53% vs. 43%).

**Conclusion:**

ILD awareness in Saudi Arabia is low (18.5%), concentrated among healthcare providers, with knowledge gaps across both the public and healthcare professionals. The persistence of perceived knowledge deficits, even among those aware demonstrates unmet educational needs. Findings support targeted public campaigns emphasising symptom recognition and primary care education to support earlier recognition, diagnosis, and timely specialist referral.

## Introduction

Interstitial lung diseases (ILDs) are a heterogeneous group of diffuse parenchymal lung disorders, including idiopathic interstitial pneumonias, connective-tissue-disease-associated ILD and sarcoidosis (1–3, 44). Recent Global Burden of Disease (GBD) analyses show that the incidence, prevalence, mortality and disability-adjusted life years (DALYs) attributable to ILD and pulmonary sarcoidosis have increased over the past two to three decade (4–6). In adults aged 50–74 years, a GBD 2021 analysis estimated more than 2.5 million prevalent ILD cases worldwide in 2021, with continued increases in age-standardised prevalence, incidence, mortality and DALY rates between 1990 and 2021 and wide geographical variation (4). Reviews from high-income settings report ILD prevalence about 0.2% and report substantial healthcare utilisation, functional limitation and impaired quality of life among affected patients (1). Idiopathic pulmonary fibrosis (IPF), the prototypical fibrotic ILD, is among the most lethal chronic lung diseases despite antifibrotic therapies, emphasising the importance of early recognition, timely diagnosis and specialist multidisciplinary care (1, 3, 7–10, 18).

In Saudi Arabia, available clinical data on ILD come mainly from small centre-based cohorts rather than population-based registries (7, 11, 43). A tertiary-centre study of 330 patients reported that connective-tissue-disease-associated ILD was the most frequent subtype, followed by idiopathic interstitial pneumonias and sarcoidosis, illustrating the heterogeneity of ILD presentations in Saudi practice (11, 43). A separate series focusing on idiopathic pulmonary fibrosis (IPF) described substantial symptom burden and poor survival among patients managed at two tertiary hospitals, underscoring the severity of fibrotic ILD in this setting (7). Despite prior contributions, population-level epidemiological data on ILD in Saudi Arabia are limited, and there is no established ILD registry capturing incident or prevalent cases at a population level (4–6, 11). Global and regional burden analyses further highlight that the Middle East and North Africa are under-represented in ILD data sources, with estimates often extrapolated from limited local cohorts rather than comprehensive surveillance systems (1, 4–6). Collectively, these gaps suggest that the true burden and phenotype distribution of ILD in Saudi Arabia are likely to be underestimated and incompletely characterised (1, 4–7, 11).

International evidence indicates that ILD is frequently diagnosed late, often after months or years of symptoms and multiple healthcare encounters (12). Early manifestations such as dyspnoea and chronic dry cough are non-specific and commonly misattributed to asthma, COPD, infection or cardiovascular disease in primary and secondary care (1, 12). Delays in recognising “red flag” features and in arranging high-resolution CT (HRCT) imaging or specialist review are associated with more advanced disease at diagnosis and worse outcomes (1, 12). Recent literature continues to emphasise that delayed recognition and fragmented referral pathways remain major barriers to timely ILD diagnosis, particularly for rare and fibrotic ILD subtypes requiring multidisciplinary evaluation (9, 10). A survey of primary care physicians across several countries found that although around two-thirds of ILD-related knowledge questions were answered correctly, only about one-fifth of clinicians felt their knowledge of ILD was satisfactory, and recognition of key clinical signs such as fine inspiratory crackles was suboptimal (13). These findings suggest that non-specialist clinicians often feel underprepared to suspect and evaluate ILD, particularly in patients with non-specific respiratory symptoms (1, 9, 10, 12, 13). In response to these challenges, international professional organisations have developed initiatives such as the CHEST “Bridging Specialties: Timely Diagnosis for ILD” programme, which calls for improved ILD awareness and education among primary care and other non-respiratory specialists to shorten time to diagnosis and facilitate earlier referral to specialist ILD centres (14). Collectively, this body of work highlights that awareness and confidence in recognising ILD are central to reduce diagnostic delays and improve outcomes (1, 12–14).

In Saudi Arabia, available evidence consistently demonstrates major gaps in public awareness of respiratory diseases, even for common conditions such as COPD (15). A nationwide survey of 15,000 adults found that approximately 77% had never heard of COPD and that overall knowledge of the disease was poor (9). Similarly, limited health literacy has been reported across the population, with 57.4% of adults demonstrating inadequate health literacy, making it difficult for many individuals to understand health information and engage with preventive care (16).

These gaps are relevant to ILD because rare or complex respiratory diseases typically require higher levels of baseline health literacy and disease familiarity for early recognition and help-seeking (1, 16). Among clinicians, studies have shown that many physicians in Saudi Arabia are unaware of or do not adhere to guideline recommendations for COPD care, reflecting broader challenges in guideline implementation and respiratory disease management (17).

Such findings suggest that ILD, being rarer, more complex and less visible than obstructive diseases is likely to be even less well recognised in Saudi Arabia (1, 15‒17). The broader rare-disease ecosystem in Saudi Arabia echoes these gaps, with national workshops and policy efforts highlighting long diagnostic delays, limited specialist services, and limited disease awareness as health-system barriers to best-practice care (19, 20). Taken together, this evidence supports the need for a national assessment to quantify ILD awareness and related knowledge gaps and to inform targeted public and professional education (15‒17, 19, 20).

## Methods

### Study design and participants

We conducted a national cross-sectional, anonymous, online survey in Saudi Arabia. Data were collected between 20 May 2025 and 31 October 2025 using SurveyMonkey. The survey link was distributed via X/Twitter, WhatsApp, institutional mailing lists, community groups. This study adhered to the STROBE guidance for observational studies and the SAMPL recommendations for statistical analysis (21). Participants provided electronic informed consent before accessing the questionnaire. No personally identifying information was collected and all analyses used de-identified data.

### Questionnaire development and KAP

The questionnaire was developed by a multidisciplinary panel (respiratory physicians, epidemiologists) using WHO KAP survey guidance and behavioural theory, including the Theory of Planned Behaviour, which links knowledge and attitudes to health-related actions. Items were adapted from existing respiratory KAP tools and ILD patient education materials, then refined through expert review and cognitive pre-testing with lay volunteers to optimise clarity and cultural appropriateness. The final instrument is consistent with WHO guidance on knowledge-attitudes-practices (KAP) surveys (26). KAP domains were defined *a priori* as follows: Knowledge (0–8) was the sum of correct responses to eight multiple-choice items; Attitude (1–5) and Practice (1–5) were calculated as the mean of Likert-scale items, with higher scores indicating more favourable attitudes and self-reported practices. Prior to survey distribution, the questionnaire underwent iterative pilot refinement to evaluate readability, comprehension, completion time, and cultural appropriateness among lay respondents from different educational backgrounds. Cognitive pretesting was performed using think-aloud feedback and item interpretation assessment to identify ambiguous wording and optimise response consistency. Minor linguistic and formatting modifications were subsequently implemented before final deployment. Internal consistency and conceptual coherence of the KAP domains were reviewed by the multidisciplinary study panel to ensure alignment with the intended constructs and survey objectives.

### Participants and data collection

Eligible participants were adults aged ≥18 years residing in Saudi Arabia who provided electronic informed consent. We analysed complete cases, defined *a priori* as respondents with all demographic items and all outcome items completed. Exclusion criteria were: (i) age <18 years, (ii) duplicate submissions (if detected), and (iii) failed response-quality checks. An attention-check item was embedded to assess response validity. Participants who did not provide responses to all demographic questions or did not pass the embedded attention-check item were excluded from the final analysis set, defining the complete cases used for statistical analysis. To improve data integrity, duplicate submissions were screened using response-pattern review and survey metadata assessment. Surveys demonstrating implausibly rapid completion time, inconsistent response patterns, or failed attention-check responses were excluded prior to analysis. The final analytic dataset therefore represented responses meeting predefined quality-control criteria. Two independent reviewers harmonised free-text responses into prespecified categories; disagreements were resolved by consensus and, if unresolved, adjudicated by a third reviewer.

### Outcome definitions

Primary outcome (ILD awareness). Prior ILD awareness was defined using the survey awareness item and dichotomised as Aware versus Unaware. Healthcare background was derived from self-reported employment sector and categorised as healthcare versus non-healthcare. The survey collected gender, age group, nationality, geographic region, educational attainment, employment sector, tobacco/nicotine use, and self-reported chronic respiratory disease status.

Awareness pathways and personal connection. Among participants classified as Aware, the survey assessed first information source, self-rated awareness level, and whether participants knew someone diagnosed with ILD.

### KAP domains

KAP domains. Knowledge (0–8) was derived as the sum of correct responses to eight multiple-choice questions. Attitude (1–5) and Practice (1–5) scores were calculated as the mean score derived from multiple Likert-scale items, with higher scores indicating more favourable attitudes and self-reported positive health practices, respectively. All three domain scores were treated as continuous variables in the regression analyses. Also, participants reported perceived barriers to seeking medical advice for possible ILD symptoms and the information topics they would like to receive; items were analysed as endorsed versus not endorsed.

### Statistical analysis

Categorical variables were summarised as counts and percentages. Comparisons between ILD awareness groups used Pearson’s *χ*^2^ test when expected cell counts were adequate; when sparse cells were present (e.g., multiple categories with expected counts <5), we used an exact test approach (Fisher–Freeman–Halton or Monte Carlo exact) or collapsed categories as prespecified. Knowledge (0–8), Attitude (1–5), and Practice (1–5) scores were compared between awareness groups overall and stratified by healthcare background using two-sided Mann–Whitney U tests; effect sizes for overall comparisons were quantified using Hedges’ g. Hedges’ g was calculated exclusively for the overall comparisons between Aware and Unaware groups.

Factors associated with prior ILD awareness were examined using multivariable logistic regression, reporting adjusted odds ratios (aORs) with 95% CIs; global *p*-values were used for multi-level predictors. Reference categories were female, age 18–24 years, Saudi nationality, Makkah region, Bachelor’s degree, and non-healthcare background. Associations of prior ILD awareness and healthcare background with KAP domain scores were evaluated using multivariable linear regression with robust (HC3) standard errors, reporting adjusted mean differences (*β*) with 95% CIs. Robust (HC3) standard errors were employed to account for potential non-constant variance in the regression residuals. Model assumptions were evaluated before final analysis. Multicollinearity was assessed using variance inflation factors, and no evidence of problematic collinearity was identified among included predictors. Residual distributions and model fit diagnostics were visually inspected to confirm the appropriateness of the regression framework. Robust HC3 standard errors were selected *a priori* to minimise the potential influence of heteroscedasticity and unequal residual variance commonly encountered in observational survey data. Models adjusted for gender, age group, nationality, region, and educational attainment; when estimating the healthcare-background association, models additionally adjusted for prior ILD awareness. To account for testing across the three KAP domains (Knowledge, Attitude, Practice) for each exposure (awareness and healthcare background), we applied the Benjamini-Hochberg false discovery rate (FDR) procedure. The resulting FDR-adjusted q-values were used to control the false discovery rate associated with multiple hypothesis testing. A secondary multivariable linear model examined independent predictors of Knowledge, using robust (HC3) standard errors and additionally adjusting for smoking status and chronic respiratory disease. All statistical tests were two-sided. For KAP domain analyses we report both nominal *p* values and Benjamini-Hochberg FDR-adjusted q values, and we interpreted domain-level statistical evidence primarily using *q* < 0.05 for the prespecified multiple-testing framework.

### Software

All statistical analyses were performed using IBM SPSS Statistics, version 31.0.1.0 (IBM Corp., Armonk, NY, USA).

### Ethical approval

Ethical approval was obtained from IRB/ethics committee, King Abdulaziz University, Jeddah, Saudi Arabia. All participants provided electronic informed consent before accessing the questionnaire.

## Results

### Characteristics of the study population

Of 830 individuals who initiated the survey, 628 (75.7%) provided complete responses. A total of 628 participants were included in the analysis, of whom 116 (18.5%) were classified as Aware of ILD and 512 (81.5%) as Unaware (Table 1). Awareness was associated with age (*p* = 0.040), academic qualification (*p* = 0.016), geographic region (*p* = 0.036), employment sector (*p* < 0.001), and chronic respiratory disease status (*p* = 0.003). The aware group included a higher proportion of participants aged 18–24 years (81.0% vs. 65.6%) and was more frequently employed in healthcare (64.7% vs. 29.5%), whereas employment in education was less frequent in the aware group (30.2% vs. 51.8% in the unaware group). Gender and nationality distributions were similar between groups (*p* = 0.178 and *p* = 0.081, respectively).

**Table 1:** Characteristics of the study population.

Variable	Category	Aware (*n* = 116) count	Aware (%)	Unaware (*n* = 512) count	Unaware (%)	*p*_value
Total participants (*N*)	Total	116	100	512	100	
Gender	Female	68	58.6	262	51.2	0.178
	Male	48	41.4	250	48.8	
Age	18–24	94	81	336	65.6	0.040
	25–29	5	4.3	28	5.5	
	30–34	2	1.7	33	6.4	
	35–39	2	1.7	38	7.4	
	40–44	5	4.3	26	5.1	
	45–49	4	3.4	25	4.9	
	50 or older	4	3.4	26	5.1	
Nationality	Saudi	112	96.6	467	91.2	0.081
	Resident (non-Saudi)	4	3.4	45	8.8	
Geographic region you live in	Makkah	73	62.9	340	66.4	0.036
	Riyadh	11	9.5	62	12.1	
	Eastern Province	12	10.3	36	7	
	Madinah	7	6	27	5.3	
	Asir	6	5.2	27	5.3	
	Qassim	0	0	7	1.4	
	Tabuk	0	0	5	1	
	Jazan	2	1.7	2	0.4	
	Al-Bahah	1	0.9	2	0.4	
	Al-Jawf	3	2.6	0	0	
	Hail	1	0.9	2	0.4	
	Najran	0	0	1	0.2	
	Northern Borders	0	0	1	0.2	
Academic qualification	Bachelor’s degree	90	77.6	315	61.5	0.016
	Elementary–middle school–high school	18	15.5	109	21.3	
	Master	3	2.6	45	8.8	
	Associate degree	4	3.4	33	6.4	
	Doctorate	1	0.9	10	2	
What is your main employment sector or field of work?	Education	35	30.2	265	51.8	<0.001
	Healthcare	75	64.7	151	29.5	
	IT & Telecommunications	2	1.7	39	7.6	
	Manufacturing	1	0.9	11	2.1	
	Transport & Logistics	1	0.9	11	2.1	
	Construction & Real Estate	0	0	11	2.1	
	Finance & Banking	1	0.9	8	1.6	
	Retail & Wholesale	1	0.9	6	1.2	
	Hospitality & Tourism	0	0	6	1.2	
	Oil, Gas & Energy	0	0	4	0.8	
Have you ever used any tobacco or nicotine products?	I have never used any nicotine or tobacco products	97	83.6	410	80.1	0.163
	I used them in the past, but I have completely quit	11	9.5	32	6.2	
	I use other tobacco products (e.g., shisha, cigars, hand-rolled cigarettes)	0	0	24	4.7	
	I currently use e-cigarettes (vapes)	3	2.6	15	2.9	
	I currently use nicotine pouches (oral nicotine sachets)	2	1.7	17	3.3	
	Multiple selections	3	2.6	14	2.7	
Do you have any chronic respiratory disease?	No	100	86.2	411	80.3	0.003
	Asthma	9	7.8	55	10.7	
	I do not know	4	3.4	46	9	
	Chronic Obstructive Pulmonary Disease (COPD)	1	0.9	0	0	
	Interstitial Lung Disease (ILD)	1	0.9	0	0	
	Pulmonary Hypertension	1	0.9	0	0	

### ILD awareness, information sources, and personal connection

Of 628 respondents, 116 (18%) reported having heard of ILD and were classified as aware, while 512 (82%) were unaware. Among aware respondents (*n* = 116), self-rated awareness was predominantly Other/unspecified (102; 88% of aware; 16% of total), with smaller proportions reporting Very aware (10; 9% of aware; 2% of total) or Not at all aware (4; 3% of aware; 1% of total).

**Figure 1:**
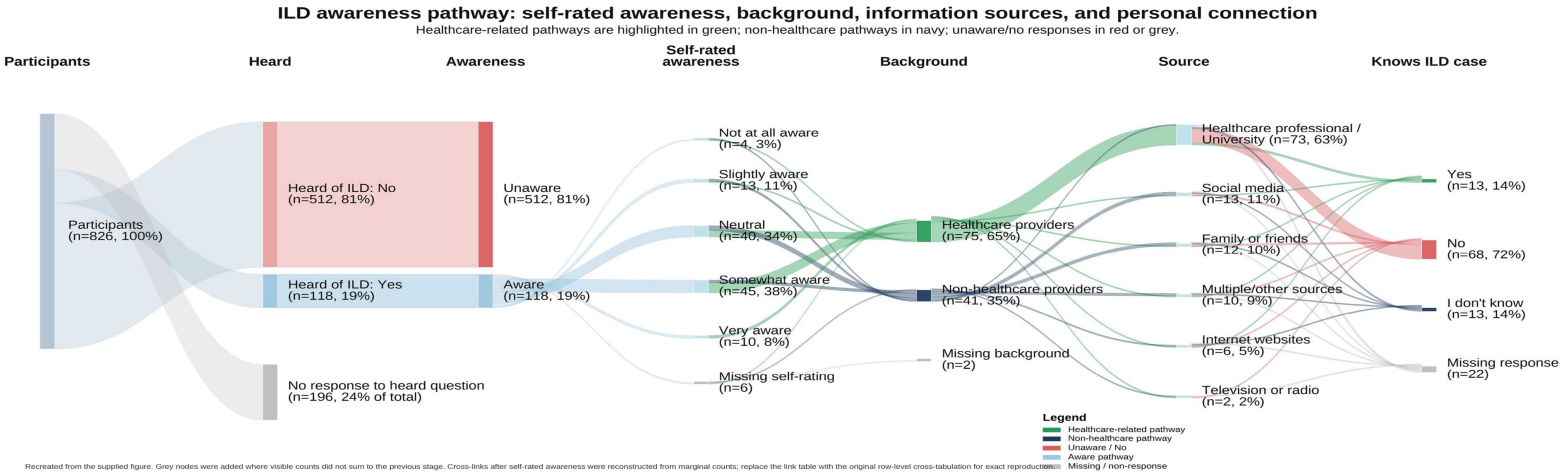
ILD awareness pathway from prior hearing to information sources and personal connection, stratified by healthcare background (*N* = 628). Sankey diagram of the ILD awareness pathway (*N* = 628). Nodes represent sequential questionnaire responses: hearing of ILD, derived awareness status, self-rated awareness, background (healthcare vs. non-healthcare), information source(s), and whether the respondent knows someone with ILD. Node labels report *n* and % of the total sample; link widths are proportional to respondent counts. The unaware branch terminates at “Unaware,” whereas the aware branch proceeds through self-rated awareness, background, information source(s), and personal connection to ILD.

Within the aware group, 75 (65%) identified as healthcare providers and 41 (35%) as non-healthcare providers. The most common source of ILD information was healthcare professionals/university (73; 63% of aware; 12% of total), followed by social media (13; 11% of aware; 2% of total), family/friends (12; 10% of aware; 2% of total), multiple sources (10; 9% of aware; 2% of total), internet websites (6; 5% of aware; 1% of total), and television/radio (2; 2% of aware; <1% of total). Most aware respondents reported not knowing someone with ILD (88; 76% of aware; 14% of total); 15 (13% of aware; 2% of total) reported knowing someone with ILD, and 13 (11% of aware; 2% of total) selected “I do not know.” (Figure 1).

### Factors associated with ILD awareness (multivariable model)

**Figure 2:**
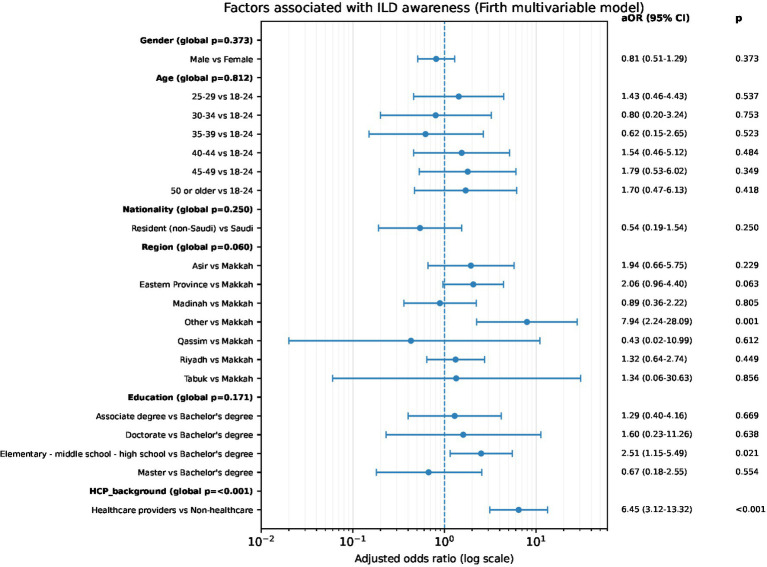
Factors associated with ILD awareness. Points show adjusted odds ratios (aORs) with 95% CIs on a log scale for ILD awareness. Reference categories were female, age 18–24 years, Saudi nationality, Makkah region, Bachelor’s degree, and non-healthcare background; dashed line indicates aOR = 1. *p* values are shown for each comparison, with global *p* values for multi-level variables.

In multivariable analysis, healthcare providers had 6.45-fold higher odds of ILD awareness compared with non-healthcare participants (95% CI: 3.12–13.32, *p* < 0.001) (Figure 2). No evidence of association was observed for gender (male vs. female: aOR 0.81 (95% CI 0.51–1.29; *p* = 0.373)), age (global *p* = 0.812 across age bands), or nationality [Resident (non-Saudi) vs. Saudi: aOR 0.54 (95% CI 0.19–1.54; *p* = 0.250)].

### Association of prior ILD awareness and healthcare background with KAP domain scores

**Figure 3:**
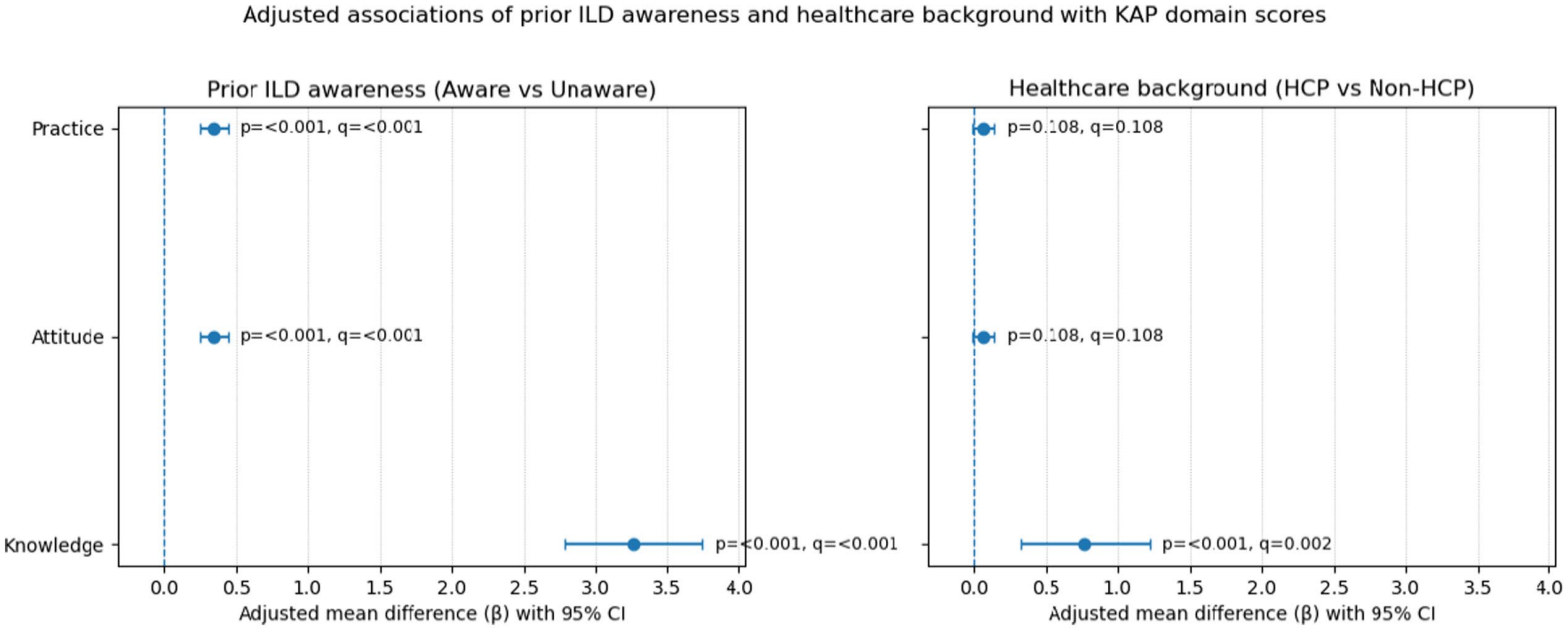
Adjusted associations of prior ILD awareness and healthcare background with KAP domain scores. Points are adjusted mean differences (*β*) with 95% CIs from multivariable linear regression (robust HC3 SE), adjusted for gender, age, nationality, region, and educational attainment; the healthcare-background model additionally adjusts for prior ILD awareness. Dashed line indicates *β* = 0; positive values indicate higher scores in the first group listed. Two-sided *p* values and FDR-adjusted *q* values are shown.

In adjusted linear regression models (robust HC3 standard errors) including gender, age, nationality, region, and educational attainment, prior ILD awareness was associated with higher scores across all three KAP domains (Figure 3). Specifically, participants who reported prior awareness had higher knowledge scores (*p* < 0.001; *q* < 0.001; Figure 3). Awareness was also associated with higher practice scores (*p* < 0.001; *q* < 0.001). Prior ILD awareness was associated with higher attitude scores in minimally adjusted models; however, in the fully adjusted models that included both prior ILD awareness and healthcare background, the association with attitude did not meet the prespecified threshold (*p* = 0.051; *q* = 0.076). After adjustment, healthcare background was associated with higher knowledge scores (*p* < 0.001; *q* = 0.002) but not with attitude or practice (both *p* = 0.108; *q* = 0.108).

### Prior ILD awareness and KAP domain scores

**Figure 4:**
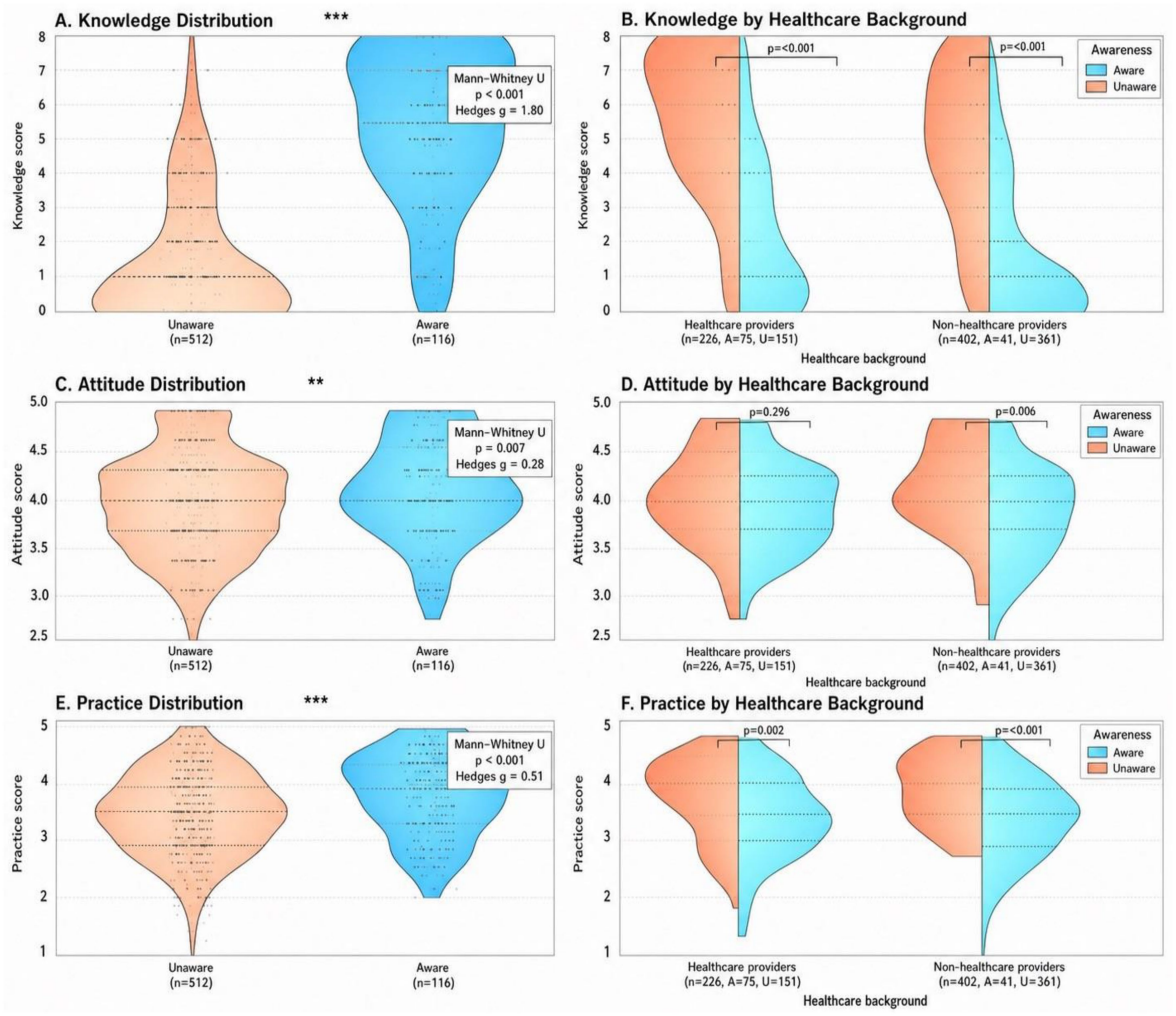
Impact of prior ILD awareness on knowledge, attitude, and practice (KAP) domain scores, overall and stratified by healthcare background. Violin plots show knowledge (0–8), attitude (1–5), and practice (1–5) scores by awareness status (aware vs. unaware). **(A,C,E)** Show overall comparisons (aware *n* = 116; unaware *n* = 512); **(B,D,F)** show split violins within healthcare background (*n* = 226; aware = 75; unaware = 151) and non-healthcare background (*n* = 402; aware = 41; unaware = 361). Points denote individuals; dashed lines indicate quartiles. Two-sided Mann–Whitney *U p* values are annotated; Hedges’ *g* is shown for overall comparisons **(A,C,E)**.

Prior awareness of ILD was associated with higher knowledge scores (0–8) compared with no prior awareness (aware *n* = 116; unaware *n* = 512; Mann–Whitney U *p* < 0.001; Hedges’ *g* = 1.80) (Figure 4A). This pattern persisted within participants with a healthcare background (*n* = 226; aware = 75; unaware = 151; *p* < 0.001) and those without a healthcare background (*n* = 402; aware = 41; unaware = 361; *p* < 0.001) (Figure 4B).

Attitude scores (1–5) were slightly higher among aware participants overall (*p* = 0.007; Hedges’ *g* = 0.28) (Figure 4C). Stratified analyses varied by healthcare background: the awareness-attitude association was not evident among participants with a healthcare background (*p* = 0.296) but remained significant among those without a healthcare background (*p* = 0.006) (Figure 4D).

Practice scores (1–5) were higher among aware participants overall (*p* < 0.001; Hedges’ *g* = 0.51) (Figure 4E), with significant differences within both healthcare background (*p* = 0.002) and non-healthcare background strata (*p* < 0.001) (Figure 4F).

Prior ILD awareness was associated with higher KAP scores. Knowledge (0–8) was higher in aware versus unaware participants (116 vs. 512; *p* < 0.001; *g* = 1.80; Figure 4A) and remained higher within both healthcare-background (75 vs. 151; *p* < 0.001) and non-healthcare strata (41 vs. 361; *p* < 0.001; Figure 4B). Attitude (1–5) was slightly higher overall (*p* = 0.007; *g* = 0.28; Figure 4C) but differed by healthcare background (healthcare *p* = 0.296; non-healthcare *p* = 0.006; Figure 4D). Practice (1–5) was higher overall (*p* < 0.001; *g* = 0.51; Figure 4E) and within both strata (healthcare *p* = 0.002; non-healthcare *p* < 0.001; Figure 4F).

**Figure 5:**
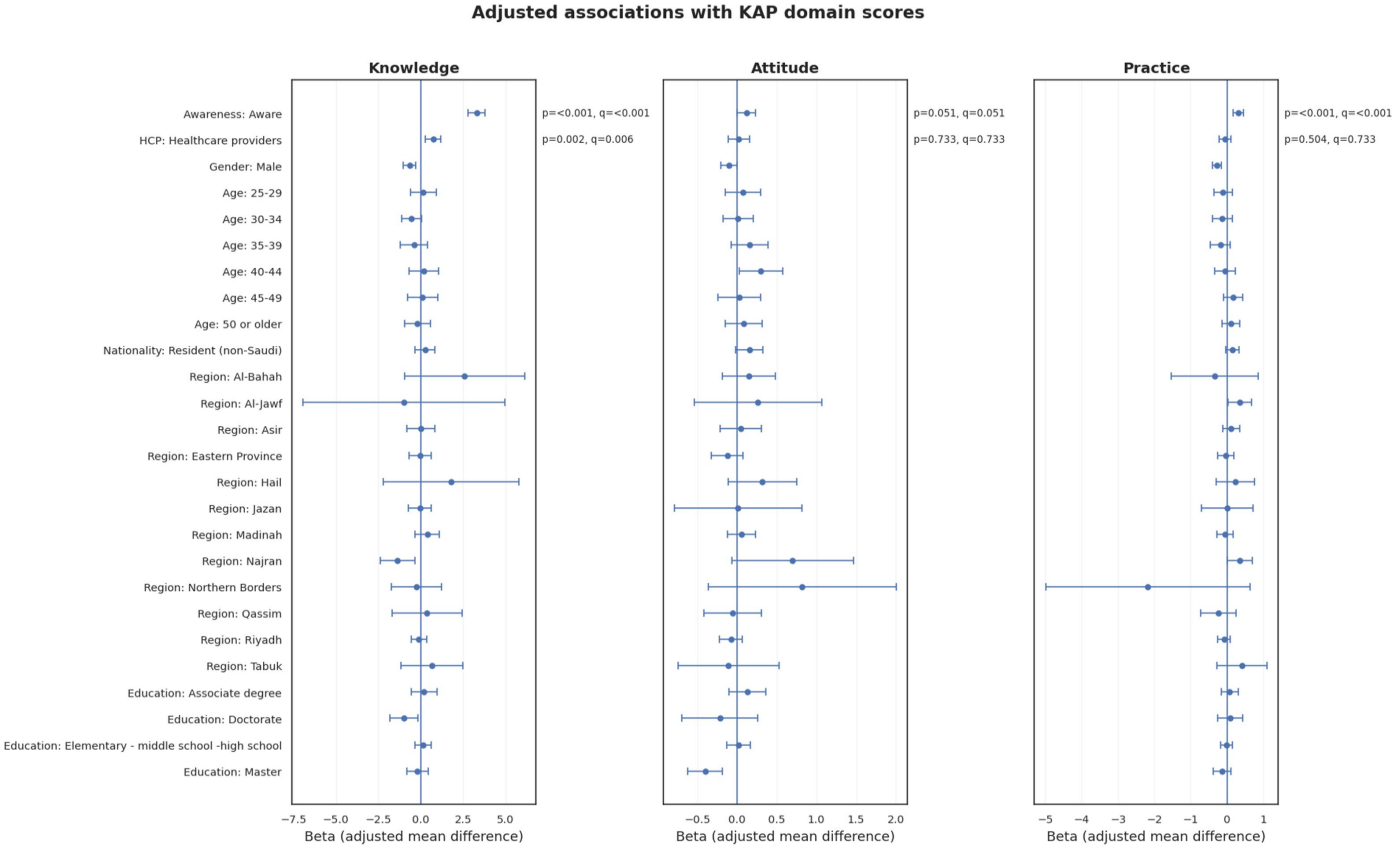
Adjusted associations with KAP domain scores. Forest plots show adjusted mean differences (β) with 95% CIs from multivariable linear regression (robust HC3 SE) for knowledge (0–8), attitude (1–5), and practice (1–5). Models include prior ILD awareness (aware vs. unaware; reference unaware) and healthcare background (healthcare vs. non-healthcare; reference non-healthcare), adjusted for gender, age group, nationality, region, and education. Vertical line indicates *β* = 0. Two-sided *p* values are shown for awareness and healthcare background; *q* values are Benjamini-Hochberg FDR-adjusted across domains for each exposure.

In covariate-adjusted linear models (robust HC3; Figure 5), awareness remained independently associated with higher knowledge and practice (both *p* < 0.001; *q* < 0.001), but not attitude (*p* = 0.051; *q* = 0.076). Healthcare background was independently associated with Knowledge (*p* = 0.002; *q* = 0.006), but not with Attitude (*p* = 0.733; *q* = 0.733) or Practice (*p* = 0.504; *q* = 0.733).

### Independent predictors of knowledge

**Figure 6:**
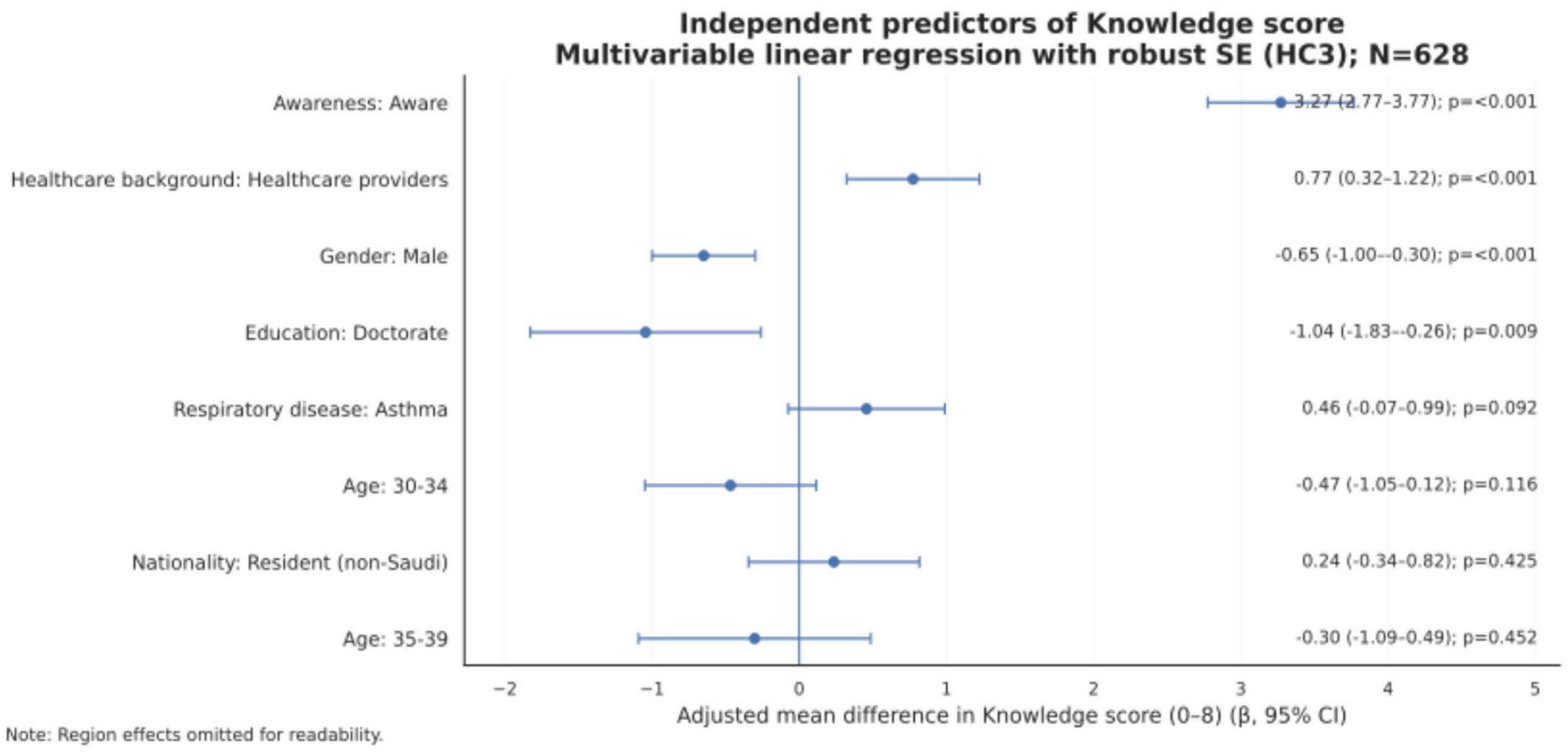
Independent predictors of knowledge (0–8). Coefficient plot from multivariable linear regression with robust (HC3) standard errors (*N* = 628). Points show adjusted mean differences (*β*) with 95% CIs (reference: unaware and non-healthcare). Models adjust for gender, age group, nationality, region, education, smoking status, and chronic respiratory disease; region terms are omitted from the plot. Vertical line indicates *β* = 0.

In multivariable linear regression with robust (HC3) standard errors (*N* = 628), prior ILD awareness was the strongest independent predictor of Knowledge (*β* = 3.27, 95% CI 2.77–3.77; *p* < 0.001; Figure 6). Healthcare background was also associated with higher Knowledge (*β* = 0.77, 95% CI 0.32–1.22; *p* < 0.001), whereas male gender was associated with lower Knowledge (*β* = −0.65, 95% CI − 1.00 to −0.30; *p* < 0.001).

### Barriers and information needs

Perceived lack of awareness remained a barrier even among those classified as aware (Unaware: 413/512, 81%; Aware: 90/116, 78%). The second most common barrier in both groups was the belief that symptoms will resolve on their own (Unaware: 261/512, 51%; Aware: 69/116, 59%).

**Figure 7:**
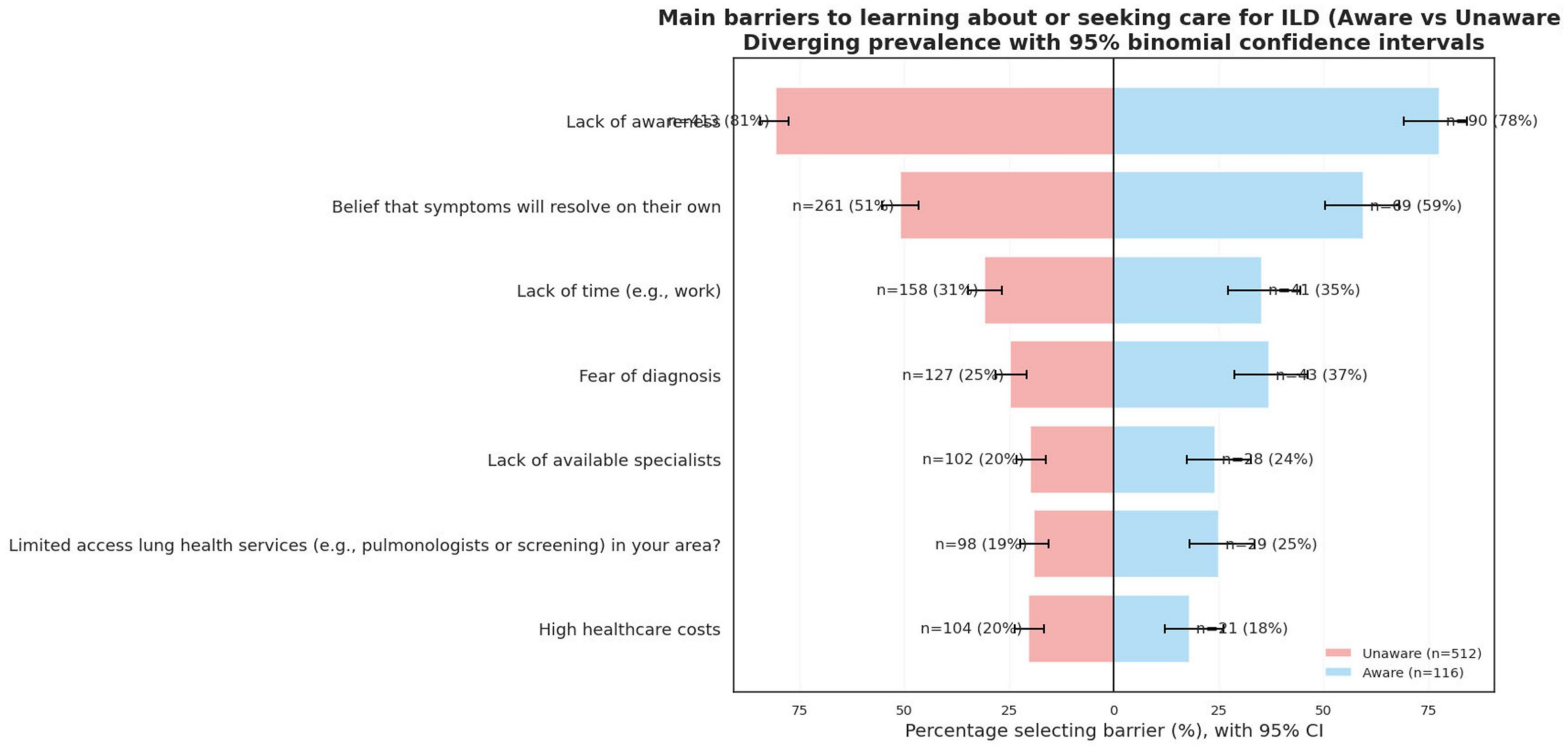
Barriers to learning about or seeking care for ILD (aware vs. unaware). Diverging bars show the % selecting each barrier (multi-select): unaware (*n* = 512; left) vs. aware (*n* = 116; right), ordered by overall prevalence. Error bars are 95% binomial CIs; labels shown (%) within each group.

**Figure 8:**
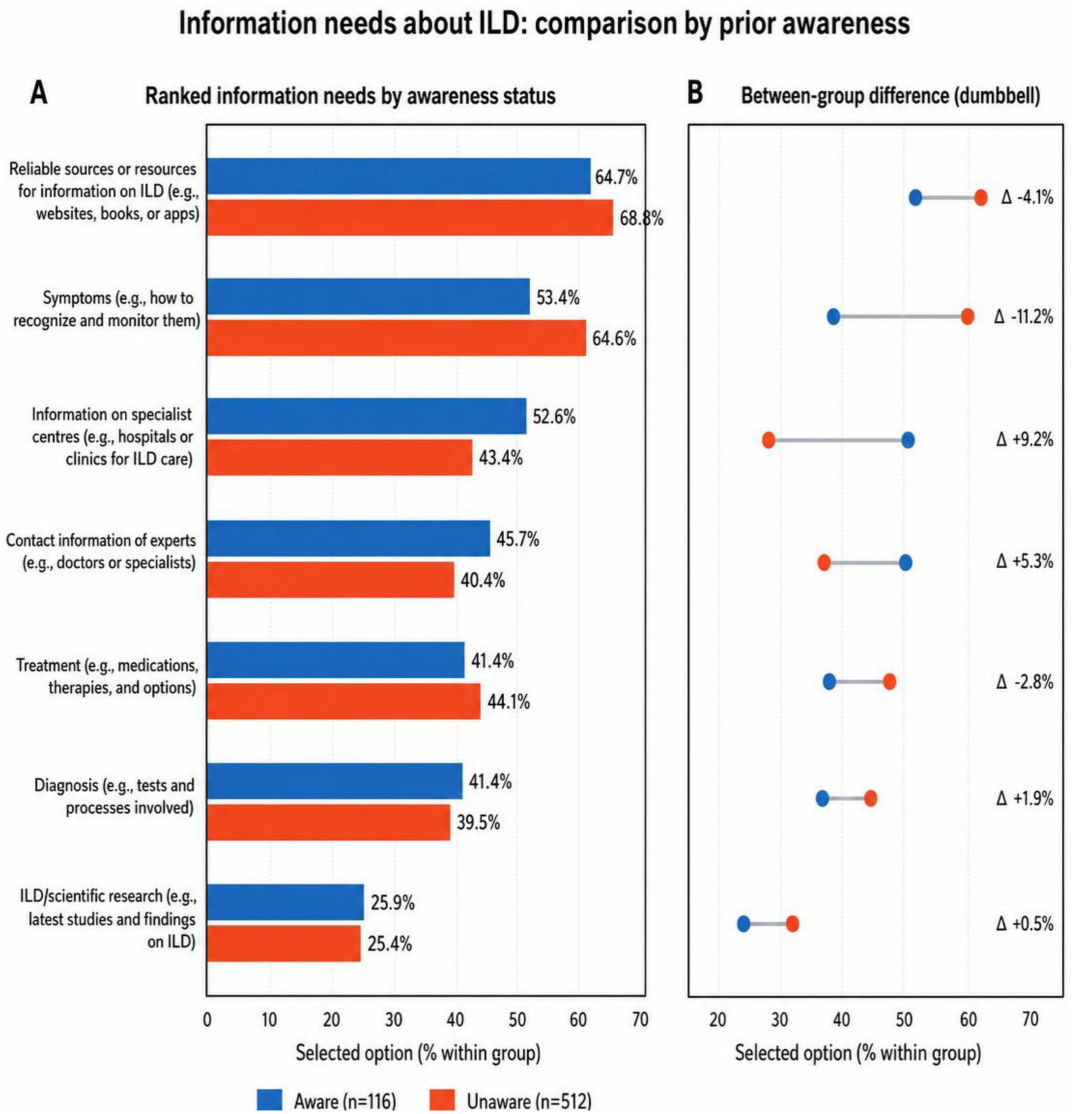
Information needs among participants aware and unaware of ILD. Panel **(A)** shows the proportion of respondents selecting each information need, stratified by prior awareness of ILD (Aware, *n* = 116; Unaware, *n* = 512). Percentages are within-group and options were multi-select (participants could select more than one need). Categories are ranked by the Aware group’s selection frequency. Panel **(B)** shows the absolute percentage-point difference between groups (Aware − Unaware) for each option; values >0 indicate higher selection among Aware participants.

Several barriers were reported more often by the Aware group than the Unaware group, including fear of diagnosis (Aware: 43/116, 37% vs. Unaware: 127/512, 25%), lack of time (e.g., work) (Aware: 41/116, 35% vs. Unaware: 158/512, 31%), and limited access to lung health services (e.g., pulmonologists or screening) (Aware: 29/116, 25% vs. Unaware: 98/512, 19%). Lack of available specialists was also slightly higher among those aware (28/116, 24%) compared with those unaware (102/512, 20%). In contrast, high healthcare costs were reported less frequently by the Aware group (21/116, 18%) than the Unaware group (104/512, 20%). Across items, the 95% binomial confidence intervals indicated substantial overlap for most comparisons, suggesting that the overall pattern of barriers was broadly similar between groups, with modest differences in specific items (Figures 7, 8).

In both groups, the most frequently selected information need was reliable sources or resources for information on ILD (e.g., websites, books, or apps) (Aware 64.7% vs. Unaware 68.8%). Among participants without prior awareness, requests clustered around practical understanding and recognition of disease: symptoms were selected by 64.6% (vs 53.4% in the Aware group; −11.2 percentage points), alongside treatment (e.g., medications, therapies, and options) (44.1% vs. 41.4%; −2.8 points). Participants with prior awareness more often prioritised information on specialist centres (e.g., hospitals or clinics for ILD care) (52.6% vs. 43.4%; +9.2 points) and contact information of experts (e.g., doctors or specialists) (45.7% vs. 40.4%; +5.3 points). Interest in diagnosis (e.g., tests and processes involved) was similar between groups (41.4% vs. 39.5%; +1.9 points).

## Discussion

We conducted the first nationwide cross-sectional survey in Saudi Arabia to evaluate public awareness of interstitial lung disease (ILD), associated knowledge-attitude-practice (KAP) levels, perceived barriers to care-seeking, and information needs. Only 18.5% (116/628) of respondents reported having heard of ILD and were classified as aware, while 81.5% were unaware. The finding is consistent with, yet more pronounced than, awareness gaps documented for other chronic respiratory conditions in the Kingdom. A nationwide survey of 15,002 Saudi adults found that approximately 77% had never heard of chronic obstructive pulmonary disease (COPD) (15), and an earlier cross-sectional study of Middle East respiratory syndrome (MERS) reported that social media was the primary information channel yet nearly 18% of the population still possessed poor knowledge of the disease (27). Taken together, these data suggest that low awareness of ILD is part of a broader pattern of limited respiratory-disease literacy in the general Saudi population, a pattern that requires targeted public-health intervention.

From a broader WHO public-health perspective, chronic respiratory diseases are one of the four major non-communicable diseases (NCDs) prioritised in the WHO Global Action Plan for the Prevention and Control of NCDs 2013–2030 (22), and the WHO Regional Office for the Eastern Mediterranean has endorsed a Regional Framework for Action built on governance, risk-factor reduction, surveillance, and health care (23). Our findings are consistent with these priorities, which emphasise surveillance, prevention, reduction of population-level risk exposures, and strengthened access to appropriate care (24). The WHO-convened Global Alliance against Chronic Respiratory Diseases further works to reduce the burden of chronic respiratory disease, with particular focus on low- and middle-income countries through surveillance, prevention, advocacy, and improved access to care (25). Although WHO respiratory programmes focus mainly on common chronic respiratory diseases such as asthma and COPD, these principles are directly relevant to ILD, where low awareness, delayed recognition, limited surveillance, and unclear referral pathways remain key barriers to earlier diagnosis and specialist care.

Awareness was strongly and independently associated with healthcare employment, higher educational attainment, younger age, and urban residence, but not with gender or nationality. Prior awareness was linked to higher knowledge (*p* < 0.001) and practice scores (*p* < 0.001) across both healthcare and non-healthcare subgroups, although the association with attitude scores was attenuated after full adjustment. Barriers to learning about or seeking care for ILD were similar between aware and unaware groups, most commonly “lack of awareness” (78–81%) and “symptoms will resolve on their own” (51–59%). Fear of diagnosis and limited access to specialists were reported more frequently by those already aware. The most pressing information needs were reliable sources (65–69%) and practical knowledge of symptoms (54–65%), with aware participants additionally prioritising specialist centres and expert contacts. This pattern is consistent with international patient and stakeholder-reported pulmonary fibrosis surveys that highlight major information deficits, unmet needs, and gaps in care pathways, including delayed diagnosis and insufficient support throughout the diagnostic period and beyond (28, 29, 33). The evidence suggests that expanding credible ILD information channels and reinforcing awareness may accelerate case identification and reduce time to ILD diagnosis (28, 30–32). Digital health approaches, including telehealth, mobile health applications, remote monitoring, and digital education platforms, may provide scalable routes to deliver reliable ILD information, support symptom recognition, and connect patients to appropriate respiratory services (47).

The independent association between healthcare background and ILD awareness. In the multivariable logistic model, healthcare providers had 6.45-fold higher odds of awareness compared with non-healthcare participants (95% CI 3.12–13.32; *p* < 0.001), and 64.7% of those classified as aware were employed in the healthcare sector. This result aligns with international evidence that familiarity with ILD remains largely confined to the medical profession. Wijsenbeek and Cottin noted that the heterogeneity of fibrotic lung diseases and the non-specific nature of early symptoms mean that even primary-care physicians frequently fail to consider ILD in the differential diagnosis (34). The INTENSITY survey in the United States further demonstrated that the typical diagnostic journey for patients with ILD is characterised by considerable delays, a median of 7 months from symptom onset to final diagnosis—and frequent misdiagnosis as asthma, pneumonia, or bronchitis (12). Our data extend these observations by showing that the awareness gap begins at the community level, where healthcare professionals possess at least foundational knowledge through their training, the lay public has almost none.

Prior awareness was associated with higher scores across all three KAP domains. The knowledge effect was very large (Hedges’ *g* = 1.80; adjusted *β* = 3.27, 95% CI 2.77–3.77; *p* < 0.001) and persisted within both healthcare and non-healthcare strata, consistent with a KAP study of ILD patients in Shanghai showing that even modest disease-relevant exposure independently predicted knowledge gains (35). In contrast to the large knowledge deficit, attitudes were resilient to unawareness. The adjusted awareness–attitude association was non-significant (*p* = 0.051; *q* = 0.076; unadjusted *g* = 0.28), with healthcare workers showing equivalent attitudes regardless of ILD awareness (*p* = 0.296) and non-healthcare participants differing only modestly (*p* = 0.006). As in Saudi COVID-19 KAP studies (36), positive attitudes appear to exist independently of specific disease knowledge, suggesting that awareness campaigns would encounter a receptive rather than resistant public (34). Practice scores were independently associated with prior awareness (*g* = 0.51; adjusted *p* < 0.001; *q* < 0.001) across both healthcare (*p* = 0.002) and non-healthcare strata (*p* < 0.001), whereas healthcare background alone was not a significant predictor (*p* = 0.504). This dissociation indicates that occupational proximity to the health system does not translate into ILD-appropriate practices without disease-specific knowledge, this is consistent with Turkish data showing knowledge and practice gaps in IPF diagnosis and management among pulmonologists (37). These findings demonstrate the need for awareness campaigns that extend beyond pulmonology to all healthcare professionals and the general public.

Analysis of self-reported barriers to learning about or seeking care for ILD revealed that perceived lack of awareness was the most commonly cited obstacle in both groups (aware: 78%; unaware: 81%), followed by the belief that respiratory symptoms will resolve spontaneously (aware: 59%; unaware: 51%). These findings mirror the diagnostic-delay literature in ILD and are consistent with recent evidence highlighting persistent delays across referral pathways, multidisciplinary evaluation, and specialist access in patients with suspected ILD (12, 31, 32). The INTENSITY survey reported that 72% of patients initially attributed their symptoms to advancing age rather than disease, leading to delayed medical consultation (12). Similarly, a Lancet Respiratory Medicine review emphasised that non-specific and insidious presenting symptoms, combined with scarce knowledge of fibrotic ILD among primary-care physicians, are among the principal causes of diagnostic delay (38). Fear of diagnosis was endorsed more frequently by aware participants (37% vs. 25%), raising the possibility that partial awareness, in the absence of adequate information, may paradoxically increase disease-related anxiety, a phenomenon described in the health-literacy literature on rare diseases, where incomplete knowledge has been shown to amplify rather than mitigate distress (39).

The information-needs analysis revealed a coherent pattern: participants without prior awareness prioritised foundational knowledge such as symptoms (64.6% vs. 53.4%) and treatment options (44.1% vs. 41.4%), whereas those with prior awareness prioritised navigational information including specialist-centre locations (52.6% vs. 43.4%) and expert contact details (45.7% vs. 40.4%). Both groups identified reliable information resources as their top unmet need (aware: 64.7%; unaware: 68.8%). These findings are consistent with a European qualitative survey of patients with IPF, in which unmet educational needs clustered around disease-progression information and access to centres of excellence (40). In the Saudi context, where ILD specialist services are concentrated in major tertiary centres, these data argue for a dual-track communication strategy, one targeting the unaware majority with basic disease literacy and symptom recognition, and another directing the partially aware towards actionable care pathways.

The low public awareness of ILD documented here carries important implications given the global trajectory of the disease burden. An age-period-cohort analysis of the Global Burden of Disease Study 2019 found that the relative risk of ILD prevalence has increased with more recent birth cohorts, and that the disease burden is rising in both men and women, albeit with differing temporal patterns (45). In the United States, ILDs were present in 0.21% of the population in 2019, contributing to 0.73% of total deaths, and crude prevalence has continued to increase (46). In Saudi Arabia specifically, connective-tissue-disease-associated ILD has been reported at notably high relative frequency—34.8% of ILD cases in one registry—compared with other regions (11). These epidemiological trends suggest that the awareness deficit identified in our study is likely to become increasingly consequential as the ILD burden grows, reinforcing the urgency of public-health education.

These findings are set against a rising national disease burden. A GBD 2021 analysis of Saudi Arabia reported that ILD age-standardised prevalence nearly doubled between 1990 and 2021, with incidence increasing by 60% and mortality by 20%, disproportionately affecting females and older adults (41). A frontier analysis of the same dataset identified Saudi Arabia among the 15 countries with the largest gap between actual and attainable ILD outcomes (4). Yet clinical data from the Kingdom remain confined to a single-centre Riyadh registry in which CTD-ILD predominated (34.8%), a proportion exceeding most European and North American cohorts (11, 41), and no population-level prevalence estimates exist (11, 42). Taken together, the rising national burden of ILD, the distinctive subtype profile observed in Saudi clinical data, and the profound lack of public awareness identified in this study support the need for a national ILD registry alongside a coordinated public-health education strategy. This study is, to our knowledge, the first to assess ILD-related KAP in the general Saudi adult population. This study include the combined evaluation of awareness, KAP, barriers, and information needs, together with robust multivariable and stratified analyses. Its limitations include the cross-sectional design, self-reported measures, possible selection bias arising from the online survey format, limited generalisability, and the simplified definition of awareness. In addition, because the survey was distributed electronically through social and institutional networks, individuals with greater internet access, higher educational attainment, or stronger health-related engagement may have been overrepresented. Although this may limit full population representativeness, online distribution enabled broad national geographic reach and facilitated inclusion of participants from multiple regions across Saudi Arabia.

Despite these limitations, the study provides the first nationwide evidence describing ILD awareness and related KAP domains within the Saudi population. The combination of behavioural assessment, multivariable modelling, and evaluation of perceived barriers and information needs offers clinically and publicly relevant insights that may help guide future respiratory-health awareness strategies, healthcare education initiatives, and national ILD policy development. Recent international evidence further highlights that improving awareness, accelerating referral pathways, and strengthening multidisciplinary ILD infrastructure remain central priorities for reducing diagnostic delay and improving long-term patient outcomes (9, 10, 31, 32).

In conclusion, more than four in five Saudi adults had never heard of ILD, yet attitudes towards respiratory health remained broadly favourable—indicating that the primary deficit is knowledge, not motivation. Healthcare background was the dominant driver of awareness, and prior awareness, regardless of source, was independently associated with higher knowledge and practice scores. With ILD prevalence in Saudi Arabia nearly doubling over three decades, these findings provide an urgent evidence base for a national awareness strategy combining public education with the establishment of a dedicated ILD registry.

## Data Availability

The original contributions presented in the study are included in the article, further inquiries can be directed to the corresponding author/s.
